# Circular Economy Research in the COVID-19 Era: a Review and the Road Ahead

**DOI:** 10.1007/s43615-023-00265-2

**Published:** 2023-03-27

**Authors:** Abderahman Rejeb, Karim Rejeb, Andrea Appolloni, Horst Treiblmaier, Mohammad Iranmanesh

**Affiliations:** 1 Department of Management and Law, Faculty of Economics, University of Rome Tor Vergata, Via Columbia, 2, Rome, 00133 Italy; 2 Faculty of Sciences of Bizerte, University of Carthage, Zarzouna, Bizerte 7021 Tunisia; 3 School of Management, Cranfield University, MK43 0AL Cranfield, Bedford, UK; 4 School of International Management, Modul University Vienna, Vienna, Austria; 5 School of Business and Law, Edith Cowan University, Joondalup, Australia

**Keywords:** Circular Economy, COVID-19, Sustainable supply chain management, Digitalization, Industry 4.0

## Abstract

The onset of the Coronavirus Disease 2019 (COVID-19) pandemic has resulted in a major crisis that has severely impacted numerous economic, environmental, and social aspects of human life. During the pandemic, the potential of the circular economy (CE) has gained increasing attention as a prospective remedy for numerous sustainability problems. This systematic literature review charts CE research in the COVID-19 era. To this end, 160 journal articles were selected from the Scopus database. The performance indicators of the literature were determined and described through a bibliometric analysis. Moreover, the conceptual structure of CE research was identified via a keyword co-occurrence network. Based on bibliographic coupling, the focus of CE research in the COVID-19 era revolves mainly around five thematic areas, including: (1) waste management; (2) digitalization and sustainable supply chain management; (3) the impact of COVID-19 on food systems; (4) sustainable development goals, smart cities, and bioeconomy; and (5) closed-loop supply chains. Overall, this review contributes to enriching the literature by determining the main thematic areas and future research directions that can help to advance the transition to the CE and reduce the impact of COVID-19 and similar disasters in the future.

## Introduction

The recent COVID-19 pandemic represents one of the most serious health crises of this decade [1, 2] that has significantly affected economic, environmental, and social facets of human well-being [3–6]. Disruptions in business activities alongside limited mobility, suspension of industrial operations, and obstacles in achieving the 2030 agenda for sustainable development are merely a few examples of COVID-19’s repercussions [7–9]. According to Liu et al. [10], the COVID-19 pandemic has urged many countries to turn to alternative resources in their manufacturing and trading of products and services due to recurrent supply chain disruptions since the spread of the virus. The pandemic has also altered the world’s operating principles, exposing the extreme lack of resilience and weakness of the prevailing economic model to adapt to unforeseen crises and shocks [1]. In the absence of clear response and mitigation strategies, the pandemic has also revealed the vulnerability of complex and over-centralized supply chains as well as the instability of world economies while exposing weak linkages across industrial sectors [5, 11–13]. As a result, COVID-19 has substantially impacted employment and increased the risk of hunger for millions owing to border restrictions and lockdowns [14, 15].

During the pandemic, the promises of circular economy (CE) initiatives concerning the current state of economic systems have gained increasing attention [16–18]. Conceptually, the CE is defined as “a change of paradigm in the way that human society is interrelated with nature and aims to prevent the depletion of resources, close energy and material flows, and facilitate sustainable development through its implementation at the micro (enterprises and consumers), meso (economic agents integrated in symbiosis), and macro (city, regions, and governments) levels” [19]. The CE concept has been hailed as an alternative to the traditional linear economy and a fundamental agenda for the development of ethical and sustainable business practices [20], and it includes various academic fields such as economic/management as well as engineering/natural sciences [21–24]. Similarly, the CE also constitutes a novel approach that aims to create a closed-loop system and minimize resource waste and emissions, thus boosting sustainability. The problems caused by global environmental damage have compelled firms to be proactive in embracing cleaner production methods and applying CE practices to maintain the value of products and materials for the longest time possible while minimizing waste generation [25]. While the CE knowledge domain is rich in terms of ideas and concepts, the current COVID-19 pandemic offers numerous options to test such principles. For instance, recent recycling and upcycling activities have fostered cooperation among organizations and consumers to slow down COVID-19 [18, 26–28]. The production of sanitizers from residual materials [29] and the manufacturing of face masks from textile remnants for healthcare facilities [30, 31] represent two CE scenarios that highlight the creation of resilient supply chains to deter COVID-19. Another CE case is the application of sustainable methods to reduce and treat medical waste as well as to purchase or recover materials locally [32]. Likewise, the necessity to protect, restore, and regenerate natural resources (e.g., energy, water, fossil fuels) has become more urgent due to food scarcity [33, 34], supply breakdown of fast-moving consumer goods, and health complications that have occurred during the pandemic [35].

Although the literature on the current state of CE research in the COVID-19 era has steadily increased, it remains widely scattered; only a few reviews have summarized this research strand to date. For example, Mahyari et al. [36] study the necessary waste management strategies to adapt to the post-COVID-19 era by covering all issues regarding the different aspects of the waste management system from generation to final disposal. Sharma et al. [37] review the impact of the COVID-19 pandemic on the progress of Sustainable Development Goals (SDGs) and describe how a green recovery stimulus, driven by CE-based solid waste management, can help to meet the stated targets of the United Nations’ SDGs. Similarly, Puertas et al. [38] conduct a systematic review of 111 articles to identify the environmental policies that can facilitate waste treatment during the COVID-19 pandemic. In the context of airports, Sebastian and Louis [39] synthesize the various aspects of waste management, including the types and sources of generated waste and practices adopted for the diverse waste streams. Finally, Liu et al. [40] use a bibliometric approach to look at the integration of the digital economy and the CE. The authors argue that the COVID-19 pandemic has ignited interest in the adoption of new technologies and CE principles.

While previous studies have enhanced the understanding of the confluence between the CE and COVID-19 and contributed to the theoretical progress of these topics, none of them have focused broadly on the CE, leaving the current literature scattered and inconclusive. The varied and evolving nature of past research in the CE field necessitates an updated review. Previous reviews have primarily relied on traditional content analysis and analyzed specific subfields of CE research (e.g., waste management), however, none of them has analyzed the entire CE field from a broader perspective. Additionally, these reviews only partially explore the potential for future research to advance CE research beyond the COVID-19 context. To fill this lacuna in research, this study provides a systematic review of CE research in the COVID-19 era. We posit that undertaking this analysis is highly important as the pandemic has highlighted the fragility of our current linear economic model, which relies on the extraction of finite resources and leads to the creation of substantial amounts of waste [41].

The CE offers a more resilient and sustainable alternative to the linear economy, in which resources are kept in use for as long as possible and waste is minimized [42, 43]. This is particularly relevant during a pandemic, as supply chains have been disrupted and the demand for certain goods has shifted, leading to a need for more adaptive and flexible economic models [44]. In addition, the COVID-19 pandemic has also brought to light the importance of environmental sustainability, as the breakdown of ecosystems and biodiversity loss have contributed to the emergence and spread of zoonotic diseases like COVID-19 [4]. The CE offers a way to address these environmental challenges and create a more sustainable future. Furthermore, the economic downturn caused by the pandemic has led to a need for more innovative and cost-effective solutions to drive economic recovery. The CE has the potential to provide these solutions, as it fosters economic growth while simultaneously reducing waste and resource depletion [2]. As a result, studying CE research in the COVID-19 era is critical for understanding the potential of this model to address the challenges posed by the pandemic and contribute to a more sustainable and resilient future.

On this basis, the main bibliometric indicators and research topics at the intersection of the CE and COVID-19 are identified, providing a general view of the themes and identifying the implications of the pandemic on this economic transition. Bibliographic coupling is employed to reveal the main thematic areas in the literature on the CE and COVID-19 and, finally, several directions for future research are proposed.

The remainder of this review is structured as follows: the research method is described in Section 2; Section 3 presents the descriptive analysis followed by the results of bibliographic coupling; Section 4 provides a detailed discussion followed by a conclusion of the main findings, limitations and potential research directions in Section 5.

## Research Method

A systematic literature review (SLR) is a well-established research method that aims to provide a comprehensive and systematic examination of existing studies in a particular field [45]. This approach involves a series of steps, including locating relevant publications, selecting the most relevant documents, evaluating their contributions, analyzing the data obtained from these documents, and synthesizing the findings. As noted by Tranfield et al. [46], a SLR represents a transparent and reproducible method for performing research, as it minimizes the potential for researcher bias [47]. However, despite its many advantages, conducting a SLR is not without its limitations. One of the main challenges associated with a SLR is the subjective nature of the process of classifying research clusters [48]. This can lead to errors and biases in the selection of articles, which impacts the validity of the results [49]. To address these limitations, the present study integrates a bibliometric analysis with the SLR approach. This integration of techniques is expected to enhance the objectivity and replicability of the review study by reducing the potential for errors and subjectivity. In their research, Colicchia and Strozzi [50] have previously demonstrated that combining SLR and bibliometric analysis can improve the accuracy and reliability of review articles. The specific steps involved in performing the SLR of CE research in the COVID-19 era are described in detail in the subsequent sections of this paper.

### Database Selection

Scopus served as the primary academic database for numerous previous review studies [51–53]. The reason behind this selection is the widespread recognition and acceptance of Scopus across the world [54]. Scopus is considered to be a very comprehensive database for articles related to a wide variety of research domains. It covers a broader range of topics compared to the Web of Science [44]. Furthermore, Scopus is explicitly recommended for research in social sciences [55].

### Publication Selection

The study starts with a search for relevant publications in the Scopus database. The researchers used various keywords related to the topic in the publications’ title, abstract, and keywords fields. The search string used for the review is the following: (“circular economy” OR “circularity” OR “closed-loop” OR “circular business model” OR “industrial symbiosis” OR “cradle” OR “CE principle*”) AND ( “covid-19” OR “pandemic” OR “coronavirus” OR “sars-cov-2” ) [44, 47]. The analysis only included articles written in English and published in peer-reviewed journals, which are considered as certified knowledge sources [56].

### Publication Evaluation

**Fig. 1:**
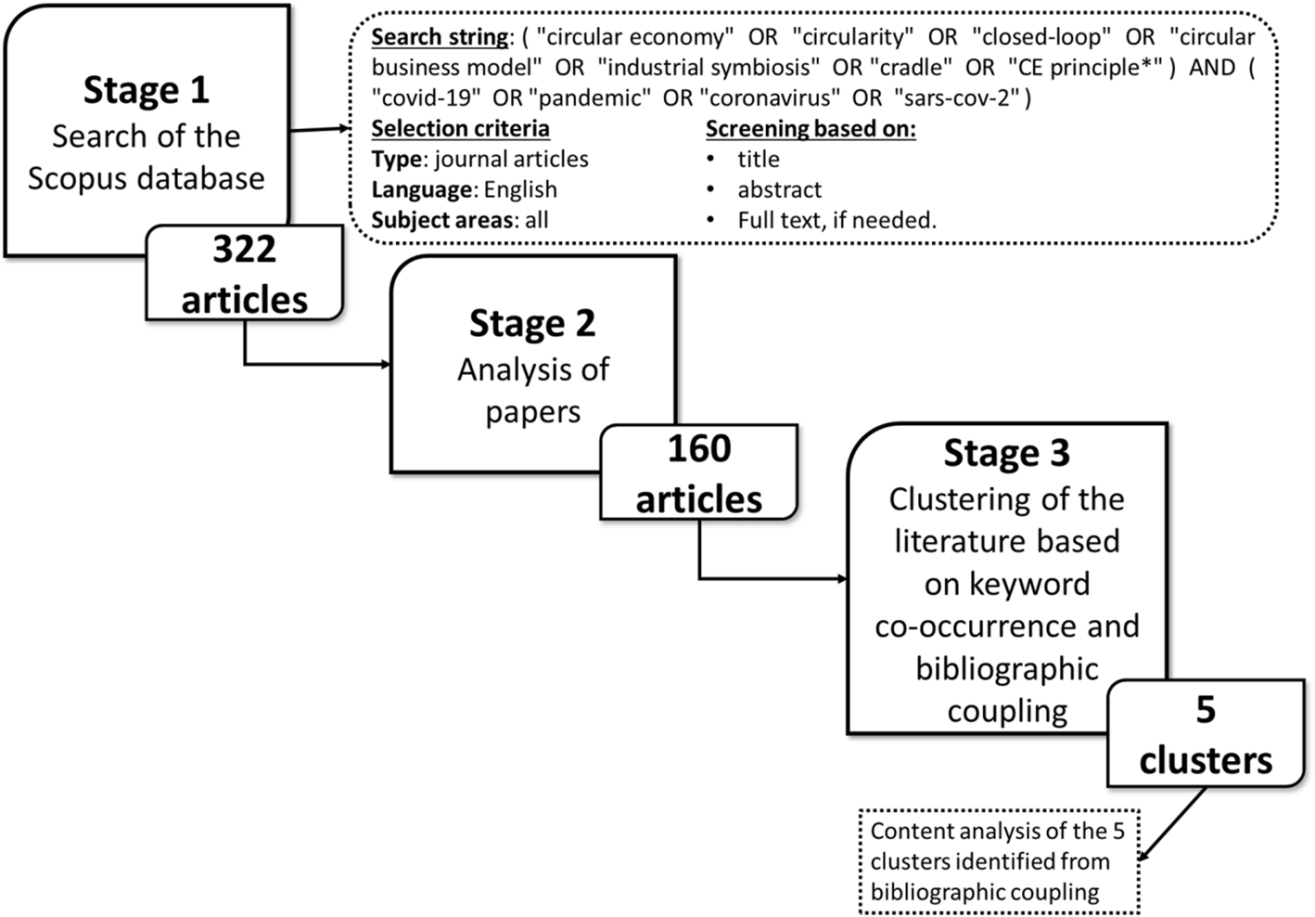
The research protocol of the SLR.

A comprehensive search was conducted in May 2022 using the Scopus database. Articles relevant to CE research in the COVID-19 era were selected using specific inclusion and exclusion criteria, which are shown in Fig. 1. The criteria included considering articles that addressed the CE, while excluding topics that do not match with the study’s scope. Next, the publications’ titles and abstracts were screened for relevance. To ensure the study’s credibility and accuracy, the authors followed the approach set forth by Aliyev et al. [57] and independently evaluated each article. Any conflicting opinions were later discussed and resolved during a group meeting. As a result, a total number of 322 relevant articles were selected for the final review.

### Bibliometric Analysis

According to Mishra et al. [58], a bibliometric analysis is a valuable quantitative method for evaluating a specific research area. This approach is particularly useful for measuring the extent of research in a field, surpassing the capabilities of SLRs. While a bibliometric analysis enhances the results of traditional literature reviews, it should not be seen as a substitute for them [59]. To further enhance the study’s thoroughness, we used VOSviewer [60] to cluster the relevant literature based on bibliographic coupling and perform a content analysis of the clustered articles. Bibliographic coupling relies on the number of common references between two publications to evaluate their mutual commonalities. Researchers can use bibliographic coupling to examine shared references between two publications to assess their similarities [61]. The larger the degree of the overlap in the publications’ bibliographies, the greater the publications’ degree of connection. Unlike co-citation analysis, bibliographic coupling does not need cumulative citations and can be applied to recent papers and emerging or less-established research fields [62]. In this SLR, we also used VOSviewer for creating a keyword co-occurrence network. This bibliometric technique helps to construct clusters that allow for a broader view of different research foci in a specific academic domain [54]. Specifically, it can enable researchers to identify the relationships between different areas of research and to study the patterns of knowledge production, dissemination, and impact. Researchers can draw on the findings of this bibliometric technique to identify the key concepts, themes, and topics in CE research and determine the relationships between these themes. The relevant literature was clustered and analyzed based on the bibliographic coupling network generated with VOSviewer. Figure 1 depicts the research protocol followed in this study.

## Findings

### Descriptive Analysis

**Fig. 2:**
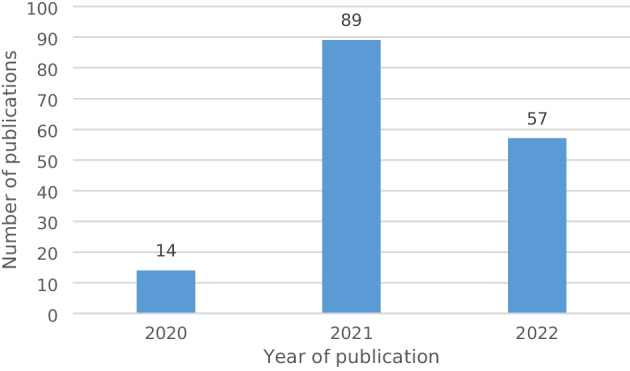
Annual evolution of circular economy research in the COVID-19 era

The period of analysis was from the beginning of 2020, when the first articles on the topic were published, to the end of May 2022. Figure 2 depicts the fluctuations and trends in the number of publications. A few articles were published in 2020, suggesting that research at the nexus of the CE and COVID-19 was a relevant topic right from the start of the pandemic [2, 37]. The scholarly output increased sixfold in 2021, which indicates the mounting interest of scholars in this research area. It is expected that the number of articles will continue to rise sharply in the near future, given that the output of the last incomplete year of 2022 has more than halved that of 2021.

Table 1 shows the journals that have published at least three articles on CE research in the COVID-19 era. Overall, these outlets published 72 papers, representing 45% of the 160 papers that were selected. *Sustainability* tops the list with 27 articles. Next is the *Journal of Cleaner of Production* with nine articles followed by *Science of the Total Environment* with six articles. The journal-wise distribution of publications suggests that CE research in the COVID-19 era was mostly published in journals that focus on sustainability and cleaner manufacturing. Moreover, the scope of the most productive journals also spans areas such as energy, waste management, logistics, and operations management. In summary, the works published in these journals indicate the diversity, interdisciplinarity, and richness of CE research in the COVID-19 era.

**Table 1:** Most productive journals (with at least three articles)

Journal	Number of articles
Sustainability	27
Journal of Cleaner Production	9
Science of The Total Environment	6
Energies	5
Environmental Science and Pollution Research	5
Resources Conservation and Recycling	4
Waste Management and Research	4
Applied Sciences	3
International Journal of Logistics Research and Applications	3
Operations Management Research	3
Waste Management	3

The affiliations of all authors were extracted to examine the geospatial distribution of the selected articles. In Table 2, we present all countries (i.e., the contributing authors’ affiliations) from which at least ten articles originate. The highest number of articles was published by authors from Italy (29) followed by China (21) and India (21). According to Giannoccaro et al. [63], Italy is among the European nations with the highest score in terms of the global circularity index. The authors note that the country has advocated for several legislative actions to facilitate the implementation of CE principles, including the Law 221/2015, which is aimed at promoting green economy and sustainable development. Financial incentives were also provided to firms to encourage the development of innovative initiatives in the CE sphere. In the transition to the CE, the Italian government has led initiatives to utilize food waste that is not fit for human consumption in the production of compost and feed. While India and the USA mostly experienced the intensity of the COVID-19 effect, Italy and China were found to have greater research endeavors to lessen the relative influence of the COVID-19 pandemic with a special focus on agrifood systems. Overall, the geographic distribution of the publications suggests that substantial research efforts at the nexus of the CE and COVID-19 have been made by researchers from both developed and developing nations.

Table 2 also lists the academic institutions that contribute the most to CE research in the COVID-19 era. The affiliation with the highest number of articles is Ilma University with five publications followed by Politecnico di Torino, Sapienza Università di Roma, and Universidad Nacional de Loja with four publications each. The list of the most productive institutions includes only a few institutions from developing countries. In general, research activities on the CE and COVID-19 are widely dispersed, with salient geographical clusters in Western countries as well as China and India [64]. This aligns with the findings from Mokuolu and Timothy [65], who note that, despite the global importance of the CE, related research remains relatively scarce in developing countries (except for China and India).

**Table 2:** Most productive countries and institutions in CE research during the COVID-19 pandemic

	Countries			Institutions		
Rank	Country	No.	%	Institution	No.	%
1	Italy	29	9.0	Ilma University	5	1.55
2	China	21	6.5	Politecnico di Torino	4	1.24
3	India	21	6.5	Sapienza Università di Roma	4	1.24
4	United Kingdom	16	4.97	Universidad Nacional de Loja	4	1.24
5	United States	16	4.97	Universiti Kebangsaan Malaysia	3	0.93
6	Spain	13	4.04	The University of Sheffield	3	0.93
7	Malaysia	11	3.42	Hanken School of Economics	3	0.93
8	Poland	11	3.42	Università degli Studi dell’Insubria	3	0.93
9	France	10	3.11	Worcester Polytechnic Institute	3	0.93
10	Iran	10	3.11	Brno University of Technology	3	0.93
11	Canada	8	2.45	Prince Sultan University	3	0.93
12	Finland	8	2.45	Universiti Teknologi Malaysia	3	0.93
13	Germany	7	2.17	Vrije Universiteit Brussel	3	0.93
14	Pakistan	7	2.17	Ural Federal University	3	0.93
15	Sweden	7	2.17	Xuzhou University of Technology	3	0.93
16	Saudi Arabia	6	1.86	Brno University of Technology, Faculty of Mechanical Engineering	3	0.93
17	Turkey	6	1.86	Beijing Key Laboratory of Urban Spatial Information Engineering	2	0.62
18	Australia	5	1.55	Loughborough University	2	0.62
19	Belgium	5	1.55	The Royal Institute of Technology KTH	2	0.62
20	Netherlands	5	1.55	Ontario Tech University	2	0.62
Total			68.94			18.94

**Fig. 3:**
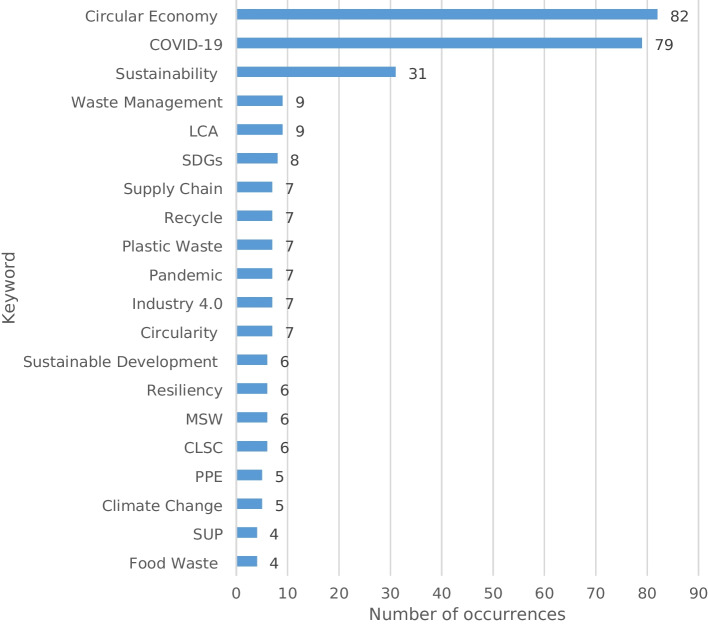
Top 20 most frequent keywords in literature

Figure 3 depicts the 20 most frequent keywords in the selected sample. According to the figure, “Circular Economy” and “COVID-19” are the most often used keywords, which is expected given that both terms were used in the search query. The third and fourth most frequently used keywords are “Sustainability” and “Waste Management.“ The recognition of sustainability is rising in modern societies due to its impact on the economic, environmental, and social aspects of human living, as summarized in the United Nation’s SDGs. Sustainability enables the reduction of waste and emissions while contributing to the creation of new economic opportunities for firms through new regulations [64]. The onset of the COVID-19 pandemic has proved that sustainability is far more than merely combating environmental hazards by also underlining the repercussions of climate change and malnutrition issues [66]. As a holistic concept, sustainability represents one of the solutions to reduce supply chain risks and uncertainties, support cleaner production systems, and promote environmentally-friendly products [67].

The COVID-19 crisis has accentuated waste generation by expanding disposable personal protective equipment (PPE) [26], municipal solid waste (MSW) [68], food waste [69], and single-use plastics (SUP) usage [70]. This development necessitates a transformation in waste management practices to slow down the plastic loop, develop intelligent product designs, and foster sustainable upcycling. The integration of CE approaches in waste management can thus help to mitigate the impact of COVID-19, support energy and material recovery, improve resource conservation, and stimulate green jobs and entrepreneurial initiatives. In this context, the term “Industry 4.0” frequently appears in the reviewed publications, revealing the enabling role of this paradigm to support smart factories [71] and optimize resource use efficiency [41]. The improvements associated with Industry 4.0 implementation in the CE include time savings in the processing of products, reduced production costs, integrated value chains, resilient manufacturing processes, and flexible and efficient resource usage [67]. Overall, the list of most frequent keywords illustrates the far-reaching potential of the CE and Industry 4.0 for reducing the impact of the COVID-19 pandemic.

The top ten articles, according to the number of citations, are listed in Table 3. Based on citations, Vanapalli et al. [72] received the highest citation number among all articles (174) followed closely by the paper from Ibn-Mohammed et al. [1] with 167 citations. These two articles have been very influential since they diffused swiftly across the CE community and laid the theoretical foundation of the pandemic’s impact on the CE in general and waste management in particular. The third position is held by Prideaux et al. [73], with 95 total citations in Scopus. This article discusses the lessons learned from COVID-19 to support the resistance of global tourism against climate change and facilitate the transition to the CE. The article by Chauhan et al. [74] received 69 citations, and the one from Del Rio Osorio et al. [69] has 42 citations. A deeper look at these publications and the remaining ones in Table 3 indicates that a key focus of past studies was on the impact of the COVID-19 pandemic on various industrial sectors, including the agrifood, healthcare, and fashion industries.

**Table 3:** Top ten most cited publications

No.	Article	Title	Journal	Citations
1	Vanapalli et al. [72]	Challenges and strategies for effective plastic waste management during and post COVID-19 pandemic	Science of the Total Environment	174
2	Ibn-Mohammed et al. [1]	A critical review of the impacts of COVID-19 on the global economy and ecosystems and opportunities for circular economy strategies	Resources, Conservation and Recycling	167
3	Prideaux et al. [73]	Lessons from COVID-19 can prepare global tourism for the economic transformation needed to combat climate change	Tourism Geographies	95
4	Parashar and Hait [74]	Plastics in the time of COVID-19 pandemic: Protector or polluter?	Science of the Total Environment	69
5	Del Rio Osorio et al. [69]	The potential of selected agrifood loss and waste to contribute to a circular economy: Applications in the food, cosmetic and pharmaceutical industries	Molecules	42
6	Chauhan et al. [75]	The interplay of circular economy with industry 4.0 enabled smart city drivers of healthcare waste disposal	Journal of Cleaner Production	41
7	Nandi et al. [2]	Do blockchain and circular economy practices improve post COVID-19 supply chains? A resource-based and resource dependence perspective	Industrial Management and Data Systems	40
8	D’Adamo and Lupi [76]	Sustainability and resilience after COVID-19: A circular premium in the fashion industry	Sustainability	34
9	Giudice et al. [16]	COVID-19, the food system and the circular economy: Challenges and opportunities	Sustainability	32
10	Vătămănescu et al. [77]	Before and after the outbreak of Covid-19: Linking fashion companies’ corporate social responsibility approach to consumers’ demand for sustainable products	Journal of Cleaner Production	30

**Fig. 4:**
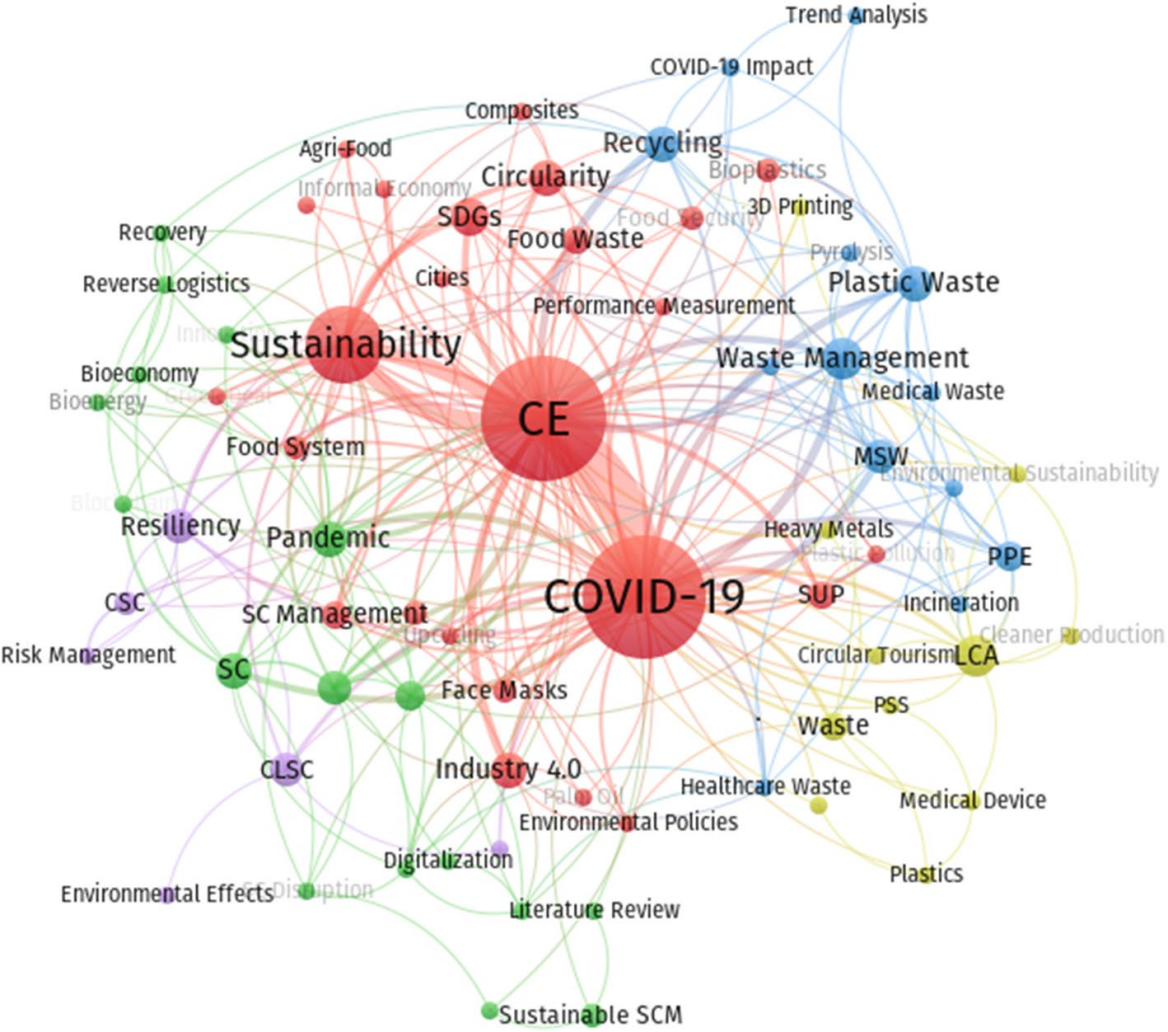
Keyword co-occurrence network

Keywords can offer the reader the primary substance of publications, and their analysis can help researchers to identify emerging research trends and hot topics in a certain knowledge field [78]. The arrangement of various research themes into clusters was visualized through the use of normalization in combination with the LinLog/modularity algorithm [79]. The edges connecting the co-occurred keywords are displayed with varying thickness, with the width of each edge representing the strength of the relationship between the keywords in question. To construct a visual representation of the co-occurrence of keywords, we made necessary adjustments and refinements to the original keywords. This involved combining similar phrases, such as “digitalisation” and “digitalization”, “blockchain” and “blockchain technology”. After cleaning and processing the data, we established a threshold of at least two occurrences for keywords in VOSviewer and generated a visual representation of the co-occurrence network. The result, shown in Fig. 4, revealed five clusters with 72 individual keywords or nodes. It can be observed from the figure that the most significant cluster is the red one in the center. The focus of this cluster is on the role of the CE in achieving sustainability and meeting SDGs in the COVID-19 era. The second cluster on the left (green color) revolves around sustainable supply chain management (SCM) and the bioeconomy. Organizations are increasingly acknowledging the importance of sustainable development and the need to develop sustainable supply chains to confront the extreme threats and uncertainties caused by the COVID-19 pandemic [80]. By adopting sustainability in the supply chain, firms can assure long-term benefits and strengthen their competitive advantages. Examples of sustainable SCM practices include the implementation of environmental management systems [80, 81], investment in green energy infrastructure [37], and the evaluation and selection of sustainable suppliers [82]. Furthermore, researchers have paid attention to the contribution of bioeconomy to the post-COVID-19 recovery by fostering economic growth, creating employment, and establishing more resilient and green energy systems [83]. The third cluster on the upper right (blue color) centers on waste management and recycling. Related keywords therefore include waste management, plastic waste, recycling, MSW, and PPE. The fourth cluster on the lower right (yellow color) was formed by 11 keywords, and it is mainly related to life cycle assessment (LCA). LCA enables the identification and measurement of the environmental impact of materials and products during the COVID-19 pandemic, including plastics [84] as well as single-use and reusable face masks [9, 85, 86]. The final cluster on the lower left (purple color) revolves around the resiliency of closed-loop supply chains. Related keywords include closed-supply chains, resiliency, circular supply chains (CSC), environmental effects, and risk management.

### Analysis of Bibliographic Coupling

**Fig. 5:**
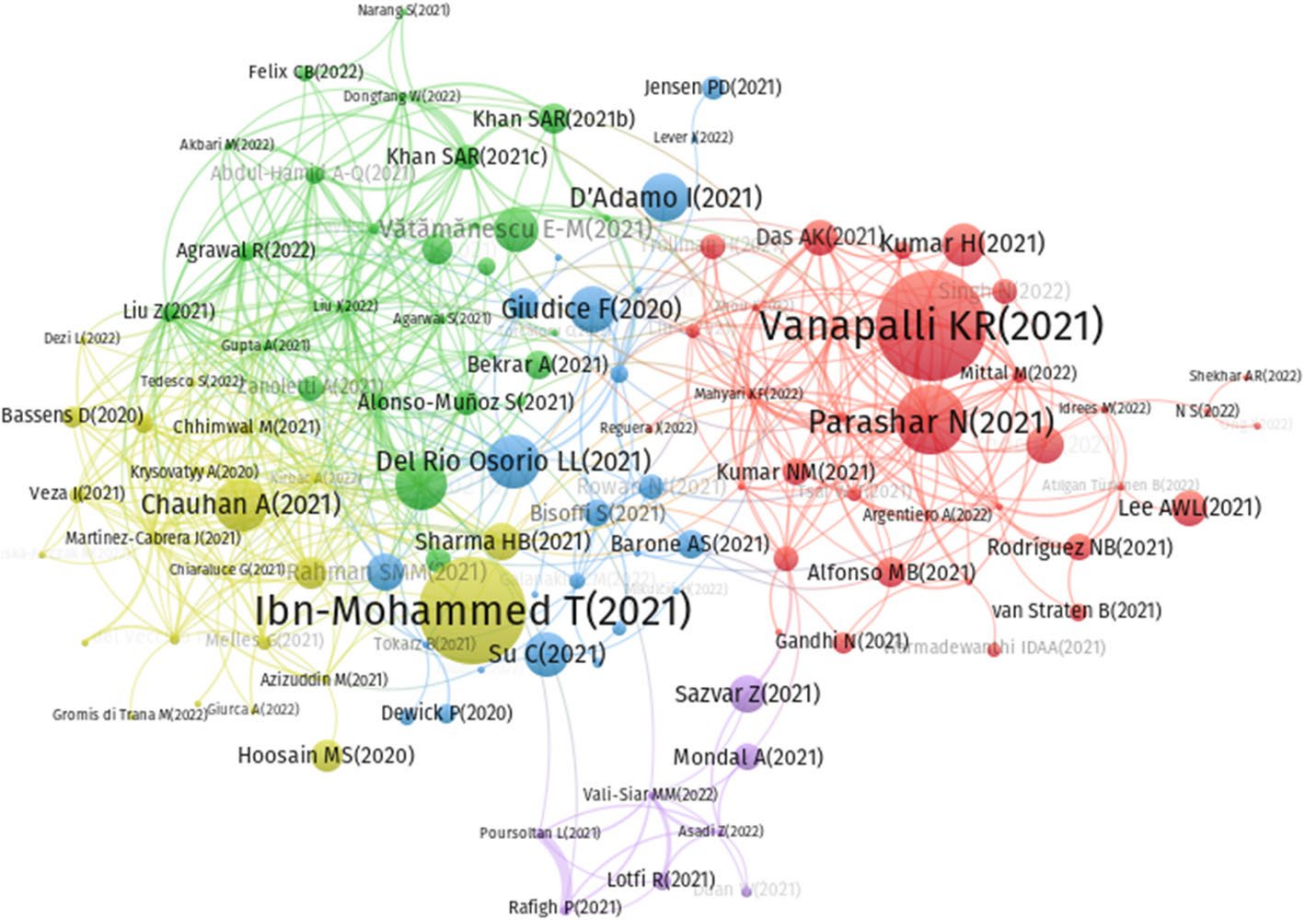
Bibliographic coupling network

To determine the key research themes pertaining to the CE in the COVID-19 era, we conducted bibliographic coupling of the selected publications using VOSviewer. The process of this technique attributed a minimum of four publications per cluster. As a result, the 160 articles generated a total of five clusters. The bibliographic coupling network is depicted in Fig. 5. Table 4 presents the top ten most cited publications in each cluster. To determine the theme of each cluster, two of the authors independently examined the titles and abstracts of the top ten influential articles to minimize any potential biases [87]. Any discrepancies were then resolved through collaborative discussion [88]. This process ensured that the themes of each cluster were accurately identified with a reduced possibility of subjective bias. Each cluster revolved around different themes: (1) waste management, (2) digitalization and sustainable supply chain management, (3) the impact of COVID-19 on food systems, (4) SDGs, smart cities, and bioeconomy, and (5) closed-loop supply chains. The findings of each cluster are discussed below.

**Table 4:** Top ten influential studies in each cluster

	Cluster red	Cluster green	Cluster blue	Cluster yellow	Cluster purple
Theme	Waste management	Digitalization and sustainable supply chain management	The impact of COVID-19 on food systems	Sustainable development goals, smart cities, and bioeconomy	Closed-loop supply chains
	Vanapalli et al. [72]	S. Nandi et al. [2]	Del Rio Osorio et al. [69]	Ibn-Mohammed et al. [1]	Sazvar et al. [89]
	Parashar and Hait [74]	Vătămănescu et al. [77]	D’Adamo and Lupi [76]	Chauhan et al. [75]	Mondal and Roy [90]
	H. Kumar et al. [9]	Khan et al. [91]	Giudice et al. [16]	Sharma et al. [37]	Lotfi et al. [92]
	Khoo et al. [93]	Jinru et al. [94]	Su and Urban [95]	D’Amato and Korhonen [96]	Duan et al. [97]
	Lee et al. [85]	Bekrar et al. [98]	Rahman et al. [99]	Hoosain et al. [100]	Rafigh et al. [101]
	Das et al. [68]	Alonso-Muñoz et al. [102]	Barone et al. [103]	Bassens et al. [104]	Vali-Siar and Roghanian [105]
	Alfonso et al. [70]	Khan et al. [67]	Bisoffi et al. [106]	Zarbà et al. [107]	Poursoltan et al. [108]
	Rodríguez et al. [86]	Su et al. [80]	Rowan and Casey [109]	Veza et al. [110]	Asadi et al. [82]
	N. M. Kumar et al. [111]	Zanoletti et al. [112]	Jensen and Orfila [113]	Melles [114]	
	Singh et al. [115]	Liu et al. [40]	Dewick et al. [116]	Chhimwal et al. [117]	
Number of articles in each cluster	34	27	24	23	8

#### Cluster 1: Waste Management

In this cluster, 34 papers deal with waste management in the COVID-19 era. Specifically, the articles discuss the impact of the pandemic on plastic and medical waste management systems. For example, Vanapalli et al. [72] offer a potential view on the disruption induced by COVID-19 on plastic waste management around the globe. According to the authors, current plastic management systems and existing infrastructure are hampered by inefficiencies in handling the flows of waste generation. As a result, there is a need for advancements in existing technologies and products that would foster economic efficiency and environmental preservation. This could be achieved, for instance, by promoting sustainable processes and products via tax rebates, limiting low recyclable plastic items, and strengthening public-private investments in research and development. Parashar and Hait [74] analyze how plastics can serve as a protector of public health and a polluter of the environment. Plastics can positively contribute to the healthcare sector during the COVID-19 pandemic if handled effectively and reinforced by CE policies in terms of recycling, recovery, and reduction, thus reducing leakage into the ecosystem. Nevertheless, the widespread perception regarding the negative impact of plastics can be confirmed owing to underutilization and mismanagement of resources, especially considering the spike in plastic consumption and waste generation resultant from the pandemic. Similarly, Khoo [93] gives insight into the situation of plastic waste before and after the COVID-19 crisis. In general, the reliance on plastics in personal protection and healthcare products has supported the fight against the COVID-19 pandemic. Although the use of PPE helped to slow down the spread of the COVID-19 pandemic, the accumulation, mishandling, and dumping of this plastic waste can result in a sudden failure of waste management systems, which may result in serious environmental degradation both on land and in the sea. Finally, Alfonso et al. [70] examine the primary COVID-19 plastic pollution dangers and offer viable methods to abate this issue. The findings of the authors imply that uniting the existing fragmented and overlapping strategies is vital to reduce plastic pollution, coordinate plastic industry laws, and foster the CE. Moreover, enhancing public risk awareness of plastic pollution is crucial to minimizing plastic waste.

Several studies are now responding to the mounting medical waste generated during the COVID-19 pandemic. For example, Kumar et al. [9] perform life cycle assessments of PPE kits under two disposal situations, notably landfill and incineration. The authors highlight that it is vital to design strategies for managing PPE waste and adopt an adequate LCA approach to support decision-making and devise sustainable strategies. Given that the COVID-19 pandemic has led to a massive production of disposable face masks, the accumulation of these items constitutes an alarming threat to the environment. In this regard, Rodríguez et al. [85] attempt to measure the environmental impact of the embedded filtration layer (EFL) reusable face masks and single-use surgical face masks. Applying LCA, the authors conclude that EFL reusable face masks have a reduced emission of at least 30% of the produced waste. Leipold and Petit-Boix [118] develop an eco-design action guide that supports the fabrication of specialized masks that can minimize the adverse impact of these products on the environment during the COVID-19 pandemic. The environmental assessment of these devices indicates that 3D-printed and washable masks are the most sustainable, helping to reduce the environmental impact and protect against the virus. While the shift to more sustainable PPE can benefit public health and environmental safety, it is still challenging to effectively manage the waste associated with these items because the generated waste is often combined with traditional waste types. For this reason, Kumar et al. [111] develop an artificial intelligence (AI) based automated system for sorting COVID-19 medical waste streams from other kinds of waste that supports data-driven decisions for recycling. Their classification model achieves a detection rate of 96.5% and enables classification of waste types within circular manufacturing. Singh et al. [115] perform a meta-analysis of healthcare and medical waste management activities in 78 nations. Their findings underline the necessity of awareness and knowledge of best practices for disease and injury prevention in relation to waste management among employees. They recommend that countries embrace environmentally sustainable management practices of healthcare waste to mitigate drastic stockpiling of virulent waste during and after health crises. Finally, beyond plastic and medical waste, Kumar et al. [119] examine issues pertaining to various municipal solid waste (MSW) management strategies. They identify potential solutions for better grasping the involved partners in waste management and offer strategies for implementation during and after COVID-19. As per the authors, the use of suitable PPE and safety measures for MSW employees is a key priority for every nation since this can lead to the generation of useful sources of energy and, consequently, sustainable development.

#### Cluster 2: Digitalization and Sustainable Supply Chain Management

29 articles contribute to the literature on digitalization and SDGs. The most influential study in this cluster is authored by Nandi et al. [2], who draw on resource-based and resource dependence theories to explore how organizations develop localization, agility, and digitization capabilities by implementing the CE and blockchain technology-related capabilities and resources. Their results demonstrate important patterns in regard to adoption degrees of blockchain-based CE systems and localization, agility, and digitization capability development. The application of blockchain can support distributed governance and automate CE processes. The wide-scale implementation of the technology can improve the traceability of products manufactured during the pandemic and allow for the development of product material passports, thereby providing detailed product footprinting and increasing compliance with environmental standards in fragmented supply chains. Similarly, Bekrar et al. [98] review practical research and issues at the confluence of transportation, reverse logistics, and blockchain. The potential advantages of the technology in transportation and reverse logistics stem from the immutability and reliability of the ledger, traceability, and smart contract utility, which acts as marketplace support and an effective mechanism for incentivization and tokenization. Besides blockchain, Khan et al. [91] posit that the harmful impact of COVID-19 can be combated with the support of technological innovation and business data analytics. The authors further find that the use of big data analytics can facilitate CE practices and enhance product delivery services, which in turn improves firm performance. Khan et al. [67] explore the interplay between Industry 4.0 technologies, the COVID-19 pandemic, environmental regulations, and CE practices. Based on a survey of 214 large and private manufacturers in Ecuador, Industry 4.0 technologies and environmental regulations are found to be the major drivers of CE practices during the pandemic. However, they found no evidence supporting the role of COVID-19 in pushing the adoption of the CE.

As the issue of resource over-consumption is currently receiving substantial attention, there is a need to achieve sustainable production and zero waste [120]. In this context, Jinru et al. [94] identify the critical role of green financing and logistics in supporting sustainable manufacturing and the CE during the COVID-19 pandemic. As an innovative technique, green financing has the potential to improve energy efficiency and accelerate the transition toward low-carbon energies [121]. According to Jinru et al. [94], green financing alongside green logistics has a substantial and positive influence on sustainable production and the CE; consequently, firms should integrate these two aspects of sustainable supply chain management into their organizational procurement and financing policies for producing sustainable products and advance CE goals. To develop resilient supply chains, Alonso-Muñoz et al. [102] employ an intellectual capital-based view and analyze the relationships between customers and suppliers from a circular supply perspective. Apart from its importance for firms, external capital can assist in establishing considerable capabilities across the entire supply chain thanks to cooperation and collaboration. In the same vein, the development of stronger and more successful relationships between customers and suppliers can bring value differentiation, competitive advantages, and improved environmental performance. This is crucial as Vătămănescu et al. [77] reveal that customers attribute great importance to environmental and social practices adopted by firms (i.e., CE issues, location of garment manufacturing, sustainable production of raw materials, emissions generated during manufacturing, protection of workers’ health and rights, and usage of recycled content) and the quality of their products.

#### Cluster 3: The Impact of COVID-19 on Food Systems

In this cluster, 22 studies contribute to a better understanding of the impact of the COVID-19 pandemic on food systems. The pandemic has been a harsh challenge for firms in the global food supply chain, exposing the pitfalls and deficiencies of food production and consumption systems. In this context, Del Rio Osorio et al. [69] systematically analyze the literature pertaining to various food loss and waste by-products, such as animal feed, that greatly contribute to the transition toward the CE. Giudice et al. [16] explore the causes and impact of COVID-19 with special emphasis on the food system. The pandemic has intensified discussions around food systems and emphasized the need for reforms in the food industry using CE solutions. For instance, the localization of food systems, environmentally-friendly food packaging, sustainable food choices, waste reduction, and bioeconomy can all contribute to more sustainable and resilient food systems [16]. By means of biodegradable packaging, Barone et al. [103] believe that food organizations can turn waste into products with significant added value and reduce their reliance on traditional nonrenewable packaging. The hygienic-sanitary advantages of these packages have also been realized during the pandemic due to increasing public awareness of the vital function of packaging for food preservation and conservation. To this end, health, sustainability, and CE considerations must be incorporated into the development of food packaging in order to reduce natural resource consumption, energy use, and waste and prioritize the creation of environmental and social values.

Bisoffi et al. [106] note that the COVID-19 pandemic provides the opportunity for reflection on the criticality of circularity in food systems, which can increase the resilience of the entire food system and its readiness for a possible future pandemic. Jensen and Orfila [113] argue that unexpected system shocks arising from disastrous events like the COVID-19 pandemic can result in food insecurity, the loss of nutritious products, and unstable food availability. As a result, developing a CE-based food ecosystem can promote the efficient use and reuse of essential nutrients, create local business opportunities, contribute to resource conservation and regeneration, and encourage environmental sustainability [109]. In this sense, Dewick et al. [116] explain that CE principles can lessen the vulnerability of small-scale farmers and guide them toward becoming a key element of formal agrifood systems, thus minimizing social and economic fragility and enhancing environmental performance.

#### Cluster 4: Sustainable Development Goals, Smart Cities, and Bioeconomy

In this cluster, researchers reflect on how the CE can contribute to the realization of SDGs, support smart city developments, and bolster the bioeconomy, thereby reducing the negative impact of the COVID-19 pandemic. For example, Ibn-Mohammed et al. [1] warn against the reliance on pandemic-driven benefits (e.g., improvements in air quality, decline in energy usage, low carbon emissions, etc.) to realize SDGs and underline the necessity of a dramatic and fundamental shift to the dynamics of existing economic systems. These include the localization of manufacturing and remanufacturing of vital medical supplies, the integration of the CE in the management of medical waste, embracing resource efficiency in the construction environment, the promotion of the bioeconomy, and the incentives and regulatory support for the CE transition. According to Zarbà et al. [107], a clear regulatory framework is required to facilitate the implementation of CE principles in the industry and to achieve environmental sustainability in the post-COVID-19 era. Sharma et al. [37] explore the effect of the COVID-19 pandemic on the progress of SDGs and offer insight into how the role of CE-based solid waste management can accomplish the targets of the United Nations SDGs. To achieve the goals of the United Nations, the transition toward the CE should be stressed in the post-COVID economic policy. While this transformation of current linear economies is challenging due to legislative, technological, and public investment difficulties, strong policies supporting supply chain localization, solid waste system decentralization, information sharing, recycling and green recovery, and global collaboration can help to achieve the SDGs [37]. The potential of technological advancements is examined in the study of Hoosain et al. [100], who argue that the fusion of Industry 4.0 technologies and the CE can help assure an inclusive and sustainable global growth that is aligned with the SDGs. Related to the theme of smart cities, Chauhan et al. [75] examine the interplay of the CE with Industry 4.0 enabled smart city drivers of healthcare waste disposal and identify several criteria for evaluating smart healthcare. The findings of the authors show that the implementation of a smart healthcare waste disposal system in the smart city is strongly motivated by the presence of digitally connected healthcare centers, waste disposal entities, and pollution control boards. Moreover, Bassens et al. [104] note that cities represent critical actors that can drive the CE agenda. Therefore, policymakers should consider the possibilities of digital CE spaces to ensure a more inclusive economy. Finally, the focus of the cluster has been placed on the need to catalyze a circular bioeconomy to support post-COVID-19 recovery efforts [122]. In this context, Veza et al. [110] examine the impact of the COVID-19 pandemic on the biodiesel industry and suggest the implementation of Industry 4.0 and the CE to overcome the challenges of COVID-19. However, D’Amato and Korhonen [96] opine that the green economy, the CE, and the bioeconomy are not sufficient solutions for prevailing economic, social, and environmental issues. Instead, the operationalization of these paradigms needs to consider global net sustainability, cascade effects, and problem displacement and shifting as well as rebound effects.

#### Cluster 5: Closed-Loop Supply Chains

The final cluster contains eight articles that discuss the importance of establishing sustainable closed-loop supply chains. For example, Sazvar et al. [89] propose a model to design a sustainable closed-loop pharmaceutical supply chain that considers the reverse flows of expired medicines, demand uncertainty, and waste management. Their results indicate that the classification of reverse flows results in efficient waste management and additional revenues as well as the reduction of disposal costs and raw material consumption. Investigating the closed-loop supply chain of ventilator devices, Asadi et al. [82] develop a multi-objective mathematical model to reduce carbon emissions as well as total costs and increase supply chain responsiveness. The authors conclude that a rise in the demand sizes of ventilators leads to environmental degradation and an increase in the overall costs while diminishing the responsiveness of the supply chain. Since logistics problems have played a critical role during the COVID-19 pandemic, it is crucial to develop a more efficient and resilient supply chain network. In this context, Mondal and Roy [90] propose an integrated sustainable production-distribution-recovery system with opened and closed-loop supply chain to optimize supply across production centers and different health institutions during the COVID-19 pandemic. Moreover, Lotfi et al. [92] examine the potential of a closed-loop supply chain to overcome demand fluctuation caused by the pandemic. Based on Lagrange relaxation, their suggested stochastic multi-objective programming model is able to estimate costs, energy use, environmental damage, and employment levels. Rafigh et al. [101] propose a new stochastic optimization model incorporating strategic and tactical decision-making to increase closed-loop supply chain responsiveness. Duan et al. [97] develop a closed-loop supply chain involving logistics and capital flows to explore the impact of the COVID-19 pandemic on producers, sellers, and recycling. Their findings demonstrate that the material flow of each primary firm in a closed-loop supply chain is more vulnerable than the capital flow during the pandemic. In addition, recyclers are identified as the primary actors heavily impacted by the material flow. Vali-Siar and Roghanian [105] propose a mathematical model to develop a resilient, responsive, and sustainable supply chain and argue that resilient policies are effective and can contribute to achieving holistic sustainability. Finally, Poursoltan et al. [108] propose a green-loop supply chain framework for ventilators and discuss a case study of Iranian medical ventilator manufacturing. The findings show that the adoption of strict policies for environmental concerns can yield considerable costs and impact carbon emissions and that demand fluctuations in the closed-loop ventilator supply chain are high in the case of a pandemic.

## Discussion

### Theoretical Implications

The shift from the linear economy to the CE has become a highly important research topic due to the growing awareness of natural resource scarcity as a result of rapid population expansion, industrialization, and persistent environmental issues [123, 124]. To help assess the evolution of the field, it is crucial to understand and structure recent developments and challenges [125]. Unlike the linear economy, the CE approach offers substantial opportunities for nations to enhance their performance in accordance with international standards on sustainable development and climate change protection. The CE also motivates organizations to gain extra value by offering secondary services and products, as well as establishing new business models without consuming additional natural resources [126].

The COVID-19 outbreak has profoundly affected people’s lifestyles and disrupted economic activities of numerous nations on a global scale. To tackle the challenges of the pandemic, considerable modifications in material flows have occurred, resulting in the uncontrolled generation of COVID-19-related waste [72]. Most of it is comprised of plastic waste that has been used in disposable medical devices, PPE, and delivery packages. In several ways, the COVID-19 pandemic has hindered the current efforts toward the CE transition and exposed the unsustainable approaches of production and consumption, which constitute the basis of the predominant linear economy system [19]. Applying bibliometric techniques, the present review examines the impact of the COVID-19 pandemic on the CE. The CE concept is crucial not only for businesses but also for the larger society and environment. Therefore, it is important to conduct a thorough research to help policymakers and organizations understand the potentials of this paradigm in addressing the challenges brought by the pandemic. This bibliometric analysis provides several theoretical insights for academics to increase their understanding of the topic. By examining the geographic coverage, yearly publication trends, sources of publications, and keywords used in previous studies, we were able to identify gaps and highlight areas for future research that can foster theoretical advancements. These findings can also assist future scholars in better understanding the current state of the CE field and formulating more effective research questions.

The findings show that the number of publications on this topic has increased significantly since 2020, and, as of May 2022, 322 papers have been published. Each of these works contributes a piece of the puzzle and provides a better understanding of CE research in the COVID-19 era. The analysis of the keyword frequency revealed most attention was paid to topics such as sustainability, waste management, life cycle assessment, and SDGs. As such, rebuilding economic systems to foster a transition toward sustainability and the realization of SDGs represent opportunities that nations must seize. Scholars also have a great interest in exploring the need for altering existing waste management practices to close the loop of plastic, food, and medical waste. Furthermore, the analysis of the keyword co-occurrence network revealed five thematic clusters that revolve around the role of the CE in fostering sustainability, the development of sustainable supply chains and bioeconomy, waste management and recycling, and the promotion of resilient closed-loop supply chains.

The bibliographic coupling network categorizes the articles published in the CE field during the COVID-19 pandemic into five primary clusters and sheds light on the role of closed-loop supply chains. First, researchers have provided insight into the limitations of current waste management systems in controlling waste generation during the pandemic and the need for CE measures and solutions to address these deficiencies. The COVID-19 pandemic has led to a significant increase in waste generation, particularly medical waste, due to the widespread use of PPE such as masks and gloves. As a result, there is a need for mechanisms of waste generation and effective waste management systems to control these flows of waste. This is crucial as current waste management systems were not designed to handle the increased volume of waste generated during a pandemic, leading to problems such as overflowing landfill sites and inadequate treatment of medical waste. To effectively and sustainably manage waste, a wide range of stakeholders, including governments, businesses, and communities, should collaborate to develop effective waste management systems [20, 26]. By working together, these stakeholders can ensure that waste management considers both the environment and society while supporting the principles of the CE.

Second, this review highlights the potential of combining digital technologies and CE practices to improve production and consumption patterns, reduce carbon emissions, and enhance workflow efficiencies. Several studies have provided valuable insights into the opportunities that digitalization and CE practices can offer for creating more sustainable and efficient supply chains in the COVID-19 era. By leveraging Industry 4.0 technologies, such as the IoT, blockchain, big data analytics, and artificial intelligence, organizations can better understand and optimize their supply chains, reduce waste, improve product quality, and enhance customer satisfaction. Additionally, digitalization can also support sustainable consumption patterns by providing consumers with greater transparency regarding the origin and environmental impact of the products they purchase. Using digital technologies to monitor and optimize energy use, organizations can reduce their carbon emissions and contribute to a more sustainable future [127]. As a result, the incorporation of digitalization can support the use of renewable energy sources, such as wind and solar power, which can further reduce the carbon footprint of supply chains.

Third, this study demonstrates that the COVID-19 pandemic has had a profound impact on food systems, exacerbating existing issues of food waste and food insecurity [44, 128]. As such, the pandemic has disrupted global food supply chains, leading to food waste in some regions and food shortages in others. Therefore, the development of circular food systems can help address these challenges by providing access to food products during pandemics, establishing effective agrifood supply chains, and reducing food loss and waste. Circular food systems are designed to create a closed-loop system of production and consumption that minimizes waste and maximizes resource utilization. By adopting circular food systems, organizations can improve the resilience and adaptability of their food supply chains, reducing their exposure to future disruptions and ensuring access to food products for consumers. Fourth, the study’s findings highlight the potential of the CE in contributing to the achievement of the Sustainable Development Goals (SDGs). They also emphasize the crucial role of CE in supporting the development of smart cities, promoting the growth of the bioeconomy and ensuring a sustainable future. In this context, future researchers can delve into the strategies required to streamline the implementation of CE principles, improve the collection and management of urban waste, and boost the development of a circular bioeconomy. Smart city developments and the promotion of bioeconomy can be key drivers for sustainable growth and resilience in the post-COVID world. As a result, the present study provides valuable guidance for policymakers, organizations, and individuals on how to leverage the potential of the CE in supporting these developments. The insights offered by our analysis can be used to create a roadmap for a more circular, resilient and sustainable future, where waste is minimized, resources are used efficiently, and economic, environmental, and social sustainability is prioritized. Finally, this review reveals that in academia mathematical optimization models are the preferred methodological approach to reduce waste, carbon emissions, total costs, energy use, and increase supply chain responsiveness and efficiency.

**Fig. 6:**
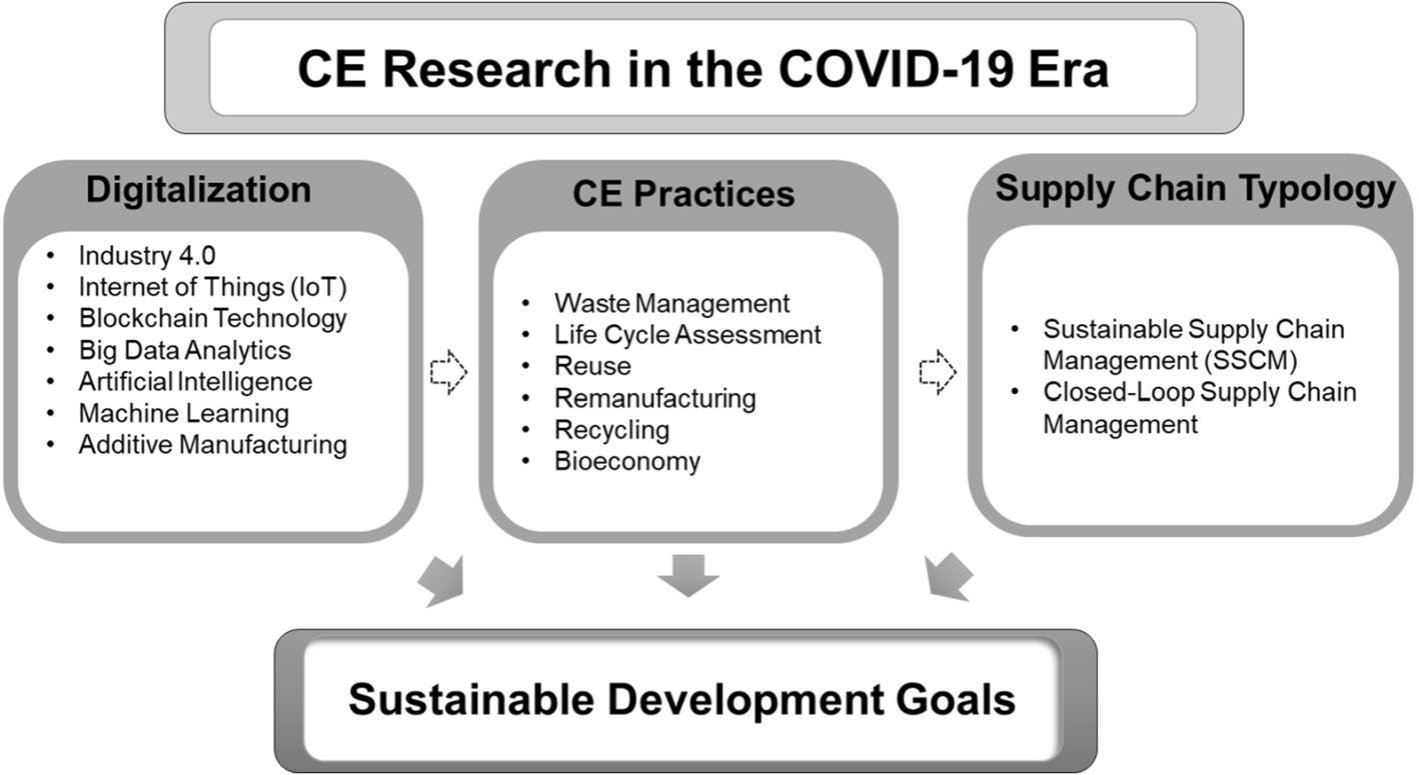
Framework for the CE in the context of the COVID-19 pandemic

Based on the insights obtained from the keyword co-occurrence and bibliographic coupling analyses, this article presents a conceptual framework for the CE in the context of the COVID-19 pandemic. The proposed framework as shown in Fig. 6 has four main components: (1) digitalization which constitutes the main focus of cluster 2 (according to bibliographic coupling) and comprises various technologies, including Industry 4.0, the IoT, blockchain technology, big data analytics, artificial intelligence, machine learning, and additive manufacturing, (2) CE practices which mainly comprise waste management, life cycle assessment, reuse, remanufacturing, recycling, and bioeconomy, and which are derived from both the keyword co-occurrence (Clusters 2, 3, and 4) and bibliographic coupling (Clusters 1, 3 and 4), (3) supply chain typology, which is developed based on the interplay of digitalization and CE practices and derived from Clusters 2 and 5 in the keyword co-occurrence and bibliographic coupling networks, respectively, and (4) SDGs which comprise the implications of integrating digitalization in the CE and developing sustainable and closed-loop supply chains. The SDGs component is mainly derived from Cluster 1 and Cluster 4 of the keyword co-occurrence and bibliographic coupling networks, respectively.

### Managerial Implications

To transition from a linear economy to a CE, managers can apply digital solutions to support the development of sustainable supply chains and bioeconomy, enhance waste management and recycling, and promote resilient closed-loop supply chains. Digitalization plays a critical role in reducing the impact of the COVID-19 pandemic by increasing automation in CE practices and driving sustainability transformation. Achieving the SDGs can be facilitated using Industry 4.0 technologies, including the Internet of Things (IoT), blockchain, big data analytics, AI, and additive manufacturing. Driven by the applications of these advanced technologies, CE actors can improve clean production strategies, develop smart manufacturing, and reduce emissions. Industry 4.0 technologies also contribute to a better utilization of repurposed and recycled products and promote safer working environments by shifting repetitive and dangerous activities from workers to machines and robots. Through digital technologies, the efficiency of recycling operations can be ameliorated while a greater proportion of recycled waste can be enhanced by sorting. Moreover, the use of digital technologies in waste collection and processing can yield a greater recovery rate [129].

To transition from the linear economy to the CE, digital solutions can also offer real-time information concerning the location, availability, and condition of materials, trace the movement of products, parts, and materials, and make the resulting information securely available. By increasing transparency and traceability, organizations can take advantage of new technologies to automatically collect material and process data for dynamic life cycle assessment analyses [130]. The integration of digitalization with LCA also improves predictive capabilities with regard to circular eco-designs and environmental sustainability, thereby increasing supply chain responsiveness and efficiency. The CE enables the efficient reuse of materials and products with a strong focus on evaluating and reducing environmental impact that might intensify climate change [131]. The implementation of CE approaches such as reuse, remanufacturing, and recycling can enhance value circulation and promote efficient resource usage, ensuring a more cost-effective and competitive post-pandemic recovery while also contributing to the reduction of greenhouse gas emissions and generating employment possibilities [37, 132]. In this regard, digitalization can help to develop products for reuse, remanufacturing, and recycling as well as decrease the cost of waste management treatment [133]. When applied in the context of pandemics and emergencies, the digital CE can support the evolution of the bioeconomy, thereby stimulating economic growth, generating employment, establishing more resilient and greener energy systems [83] as well as exerting a positive social impact [134]. As a result, through the combination of digitalization and CE practices, stakeholders can develop more efficient, resilient, and sustainable closed-loop supply chains that can be a pillar for the success of the SDGs.

## Conclusion, Limitations, and Future Research

This research presents the findings from a SLR of CE research in the COVID-19 era, including a bibliometric analysis of 160 peer-reviewed papers in the Scopus database. The performance of CE research during the pandemic was analyzed to provide a better understanding of the evolution of scholarly research, the most relevant journals, the most productive countries and institutions, and the most influential publications. Moreover, keyword frequency analysis, keyword co-occurrence network analysis, and bibliographic analyses revealed the conceptual and intellectual structure of CE literature. This review provides academics and practitioners with several important insights and implications. Despite the growth of scholarly research on the effects of COVID-19 on the CE, comprehensive studies on the topic are still very scarce, and there are several aspects to be investigated to form a complete picture of the CE that extends even further than the COVID-19 pandemic. Based on the clusters obtained from the bibliographic coupling, we propose several directions for future research in Table 5.

**Table 5:** Suggestions for future research

Cluster	Suggestions for future research
1) Waste management	• Study how waste recovery issues vary between consumer products [135] • Propose alternative waste treatment procedures to lessen the environmental impact of products and materials during pandemics [39] • Develop solutions to reduce the safety and health risks for employees who work in the waste management sector [68] • Examine holistic approaches and appropriate technological adaptations to accelerate the incorporation of waste management into the CE [115]
2) Digitalization and sustainable supply chain management	• Conduct studies focused on the drivers and challenges of Industry 4.0 technologies during and after pandemics [41, 133, 136] • Explore the relationship between Industry 4.0 technologies in the context of the CE and COVID-19 [67] • Examine the impact of circular supply chains on social sustainability [41, 77] • Propose frameworks for improving the viability of sustainable supply chains during and after the COVID-19 pandemic [137]
3) The impact of COVID-19 on food systems	• Develop effective solutions to improve food security during emergencies and pandemics [106, 128] • Investigate the opportunities and challenges of developing circular food supply chains • Increase research in materials science and engineering to design sustainable food packaging solutions [16] • Assess the impact of CE principles on each stage of the agrifood supply chain
4) Sustainable development goals, smart cities, bioeconomy	• Explore the feasibility of incorporating CE practices into the organization to meet SDGs [37] • Discuss the status of SGD initiatives and CE practices across countries during and after the COVID-19 pandemic [64] • Examine the potential of smart cities for the development of CE innovations and the realization of SDGs [75] • Discuss how circular bioeconomy can accelerate the transition toward an inclusive, renewable, and neutral economy [110, 122]
5) Closed-loop supply chains	• Examine the closed-loop supply chain design considering economic, environmental, and social sustainability [82] • Explore the impact of closed-loop supply chain practices and Industry 4.0 technologies on company performance • Develop benchmark models for supply chain members to effectively conduct and manage closed-loop supply chain strategies and sustainable targets [89] • Investigate the role of horizontal and vertical collaboration in closed-loop supply chains to reduce emissions and costs [102]

This study presents a detailed bibliometric analysis that aids in identifying, organizing, enclosing, and examining essential elements of the subject while also emphasizing the need for additional research. The present study provides the performance results of the research at the intersection of the CE and COVID-19, considering the most impactful contributors (i.e., journals, countries, and academic institutions) and relevant themes. The review enriches the current CE literature on the impact of the COVID-19 era and the need to raise CE awareness among practitioners and decision-makers. The study’s findings assist researchers in gaining a thorough knowledge of worldwide research conducted on the CE in the COVID-19 era and how it is distributed among journals, nations, and academic institutions. Furthermore, it helps scholars to obtain an understanding of the origins, development, and present state of CE research as well as reveal the most important trends in the field and identify potential research pathways.

Despite its significant contributions, this review has some limitations. Due to the timing of the onset of the COVID-19 pandemic, we only analyzed publications from the past two years, starting with the emergence of the pandemic (2020–2022). Consequently, future studies may contest or validate the findings of this review by undertaking a comparable investigation within a reasonable time period. In addition, the keywords used in the search were based on the original body of literature. Any new collection of keywords may provide different insights that might enrich the field’s rising trends. Moreover, using only the Scopus database for this review might have led to the oversight of important literature. Hence, future studies may expand this review by considering additional scientific databases. In summary, this review demonstrates that the thematic structure of the area spans several industrial sectors and is continuing to expand. As a result, scholars from different disciplines need to contribute to CE research by taking a multidisciplinary perspective. Finally, it is suggested that active academic institutions organize further academic events to foster communication and debate between academics and practitioners.

## Data Availability

Not applicable.
