# Correction to: Circular Economy Research in the COVID-19 Era: A Review and the Road Ahead

**DOI:** 10.1007/s43615-023-00266-1

**Published:** 2023-04-03

**Authors:** Abderahman Rejeb, Karim Rejeb, Andrea Appolloni, Horst Treiblmaier, Mohammad Iranmanesh

**Affiliations:** 1 Department of Management and Law, Faculty of Economics, University of Rome Tor Vergata, Via Columbia, 2, 00133 Rome, Italy; 2 Faculty of Sciences of Bizerte, University of Carthage, Zarzouna, 7021 Bizerte, Tunisia; 3 School of Management, Cranfield University, Cranfield, Bedford, MK43 0AL UK; 4 School of International Management, Modul University Vienna, Vienna, Austria; 5 School of Business and Law, Edith Cowan University, Joondalup, Australia

**Correction to** **:** **Circular Economy and Sustainability**

**https://doi.org/10.1007/s43615-023-00265-2**

The original article has been corrected as the names of the last two authors were mistyped. The names of the last two authors were corrected from Mohammad Treiblmaier and Mohamad Iranmanesh to Horst Treiblmaier and Mohammad Iranmanesh

